# Supplementary material for: Impact of Delayed Intravitreal Anti-Vascular Endothelial Growth Factor (VEGF) Therapy Due to the Coronavirus Disease Pandemic on the Prognosis of Patients with Neovascular Age-Related Macular Degeneration
Source: J Clin Med. 2022 Apr 21;11(9):2321. doi: 10.3390/jcm11092321 (PMC9100166; doi:10.3390/jcm11092321)
Supplement: Supplementary file 1 [file jcm-11-02321-s001.zip › jcm-1632698-supplementary.pdf]

Table S1. The inclusion and exclusion criteria.

Inclusion Criteria	Exclusion Criteria
nAMD patient aged ≥ 50 years	AMD with a disciform scar or geographic atrophy
Completed three initial monthly intravitreal anti-VEGF injections	Did not require intravitreal anti-VEGF injection due to the lack of disease activity
At least one delayed intravitreal anti-VEGF injection between February and June 2020	High refractive errors more than 4.0 D
The delay period is more than the number of weeks equal to the number of average months of follow-up (e.g., ≥2 weeks for patients whose average follow-up interval was 2 months before)	Diseases that could affect anatomical structure of the retina (e.g., diabetic retinopathy, retinal vein occlusion, etc.)
Never been late for a medical appointment before February 2020	History of intraocular surgery except for cataract surgery

nAMD, neovascular age-related macular degeneration; AMD, age-related macular degeneration, VEGF, vascular endothelial growth factor; D, diopter.
